# Integrated miRNA-mRNA Analyses of Triple-Negative Breast Cancer in Black and White Patients with or Without Obesity

**DOI:** 10.3390/ijms26189101

**Published:** 2025-09-18

**Authors:** Fokhrul Hossain, Martha I. Gonzalez-Ramirez, Jone Garai, Diana Polania-Villanueva, Li Li, Farzeen Nafees, Md Manirujjaman, Bolin Liu, Samarpan Majumder, Xiao-Cheng Wu, Chindo Hicks, Luis Del Valle, Denise Danos, Augusto Ochoa, Lucio Miele, Jovanny Zabaleta

**Affiliations:** 1 Department of Genetics, Louisiana State University Health Sciences Center, New Orleans, LA 70112, USA; mmanir@lsuhsc.edu (M.M.); smaju1@lsuhsc.edu (S.M.); chick3@lsuhsc.edu (C.H.); lmiele@lsuhsc.edu (L.M.); 2 Center for Biomedical Informatics and Genomics, School of Medicine, Tulane University, New Orleans, LA 70112, USA; mgonzalezramirez@tulane.edu; 3 Stanley S. Scott Cancer Center, Louisiana State University Health Sciences Center, New Orleans, LA 70112, USA; jgarai@lsuhsc.edu (J.G.); dpolan@lsuhsc.edu (D.P.-V.); lli@lsuhsc.edu (L.L.); 4 Department of Computer Science, University of New Orleans, New Orleans, LA 70148, USA; fnafees@uno.edu; 5 Department of Interdisciplinary Oncology, Louisiana State University Health Sciences Center, New Orleans, LA 70112, USA; bliu2@lsuhsc.edu (B.L.); ddanos@lsuhsc.edu (D.D.); aochoa@lsuhsc.edu (A.O.); 6 Louisiana Tumor Registry and School of Public Health, Louisiana State University Health Sciences Center, New Orleans, LA 70112, USA; xwu@lsuhsc.edu; 7 Department of Pathology, Louisiana State University Health Sciences Center, New Orleans, LA 70112, USA; ldelva@lsuhsc.edu

**Keywords:** breast cancer, TNBC, African American, microRNA (miRNA), miRNA-seq, mRNA-seq

## Abstract

Triple-negative breast cancer (TNBC) is an aggressive, heterogeneous subtype of breast cancer. miRNAs play an essential role in TNBC pathogenesis and prognosis. Obesity is linked with an increased risk for several cancers, including breast cancer. Obesity is also related to the dysregulation of miRNA expression in adipose tissues. However, there is limited knowledge about race- and obesity-specific differential miRNA expression in TNBC. We performed miRNA sequencing of 48 samples (24 tumor and 24 adjacent non-tumor tissues) and RNA sequencing of 24 tumors samples from Black (AA) and White (EA) TNBC patients with or without obesity. We identified 55 miRNAs exclusively associated with tumors in obese EA patients and 33 miRNAs in obese AA patients, each capable of distinguishing tumor tissues from obese from lean individuals within their respective racial groups. In EA, we detected 41 significant miRNA–mRNA correlations. Notably, miR-181b-5p and miR-877-5p acted as negative regulators of tumor-suppressor genes (e.g., *HEY2*, *MCL2*, *HAND2*), while miR-204-5p and miR-143-3p appeared to indirectly target oncogenes (e.g., *RAB10*, *DR1*, *PTBP3*, *NCBP1*). Among AA patients, we found 28 significant miRNA–mRNA interactions. miR-195-5p, miR-130a-3p, miR-130a-5p, miR-424-5p, miR-148a-3p, miR-374-5p, and miR-30a-5p each potentially downregulated two or more genes (e.g., *CLCN4*, *PLCB1*, *CDC25B*, *AEBP2*, *ERBB4*). Pathway enrichment analysis highlighted *KRAS*, *ESR1*, *ESR2*, *RAB10*, *TNRC6C*, and *NCAN* as the most commonly differentially expressed in EA, whereas *ERBB4*, *PLCB1*, and *SERPINE1* were most frequently in AA. These findings highlight the importance of considering race-specific miRNA–mRNA signatures in understanding TNBC in the context of obesity, offering insights into biomarker-driven patient stratification for targeted therapeutic strategies.

## 1. Introduction

Triple-negative breast cancer (TNBC) is a subtype of breast tumor that represents nearly 10–20% of all invasive breast cancers (BCs) and is characterized by deficient expression of the estrogen receptor (ER), progesterone (PR), and the human epidermal growth factor receptor 2 locus (HER2−) [1,2,3,4,5]. Due to its more aggressive clinical course and limited effective therapeutic targets, patients with TNBC have a worse prognosis than those diagnosed with other BC subtypes [6]. Clinically, TNBC is associated with a high recurrence rate and increased metastasis, leading to higher mortality rates and poorer overall prognosis than other BC subtypes. TNBC typically manifests as high-grade tumors, is often diagnosed at advanced stages, and disproportionally affects younger patients, especially African American (AA) women [7,8]. In addition, current treatments for metastatic BC, such as HER2 receptor antagonists and hormone therapy, are ineffective against the TNBC subtype [9]. Amongst limited targeted treatment options, cytotoxic chemotherapy remains the primary treatment approach for TNBC patients [10]. Therefore, there is an urgent need for identifying novel biomarkers and developing targeted therapies. MicroRNAs (miRNAs) have been implicated in several human cancers and, therefore, have been studied for their potential use as novel biomarkers with diagnostic, predictive, and prognostic potential due to their accessibility, high specificity, and sensitivity [11].

miRNAs are a class of conserved, non-coding RNAs that regulate gene expression post-transcriptionally, affecting over 60% of human genes [12,13,14,15,16]. They modulate key physiological processes, such as cell growth and disease progression, and have been implicated in complex diseases, including BCs [17,18]. Given their dual role as oncogenes (oncomirs) or tumor suppressors, miRNAs are considered promising molecular targets for cancer therapy [19,20]. Dysregulation of miRNA expression has been linked to TNBC development, metastasis, and chemoresistance [21,22,23]. For instance, altered expressions of specific miRNAs have been associated with TNBC resistance to doxorubicin and changes in malignant behavior [24,25]. These findings highlight the critical role of miRNAs in TNBC pathogenesis and prognosis, underscoring the need for further investigation [26,27].

Obesity is associated with an increased risk for several types of cancer, including breast cancer [28,29,30]. Obesity, as an inflammatory stimulus, plays an important role in breast cancer progression. Recent clinical evidence also supports the impact of body mass index (BMI) on treatment outcomes. A post hoc analysis of the phase III GIM2 trial [31] showed that BMI significantly influenced the efficacy of adjuvant chemotherapy schedules in breast cancer patients. Differential expressions of miRNAs in patients with normal weight or obesity have also been reported [32,33,34,35,36]. Ibarra et al. summarized in a review paper that obesity is associated with the dysregulation of miRNA expression in adipose tissue, which is responsible for various metabolic alterations, including the development of insulin resistance [37]. In addition, race-specific miRNA profiling has been conducted on tumor vs. adjacent non-tumor tissues of Black and White patients in a wide array of carcinomas, including breast [38], endometrial [39], ovarian [40], kidney [41], thyroid [42], and prostate [43]. In parallel, recent genomic studies have highlighted broader racial disparities in breast cancer biology, with differences observed in ctDNA profiles, therapy use, and clinical outcomes among metastatic cases [44]. However, to date, there is limited information about the race-specific differential miRNA expression in TNBC based on obesity status. Numerous recent studies have highlighted the role of miRNA in aggressive TNBC phenotype, prognosis, and survival of TNBC patients [45,46,47]. However, one limitation of miRNA studies is the variability in identified miRNA signatures across different publications. While some reports identify a single miRNA as a prognosis or diagnostic marker for breast cancer, others reported multiple miRNAs. Therefore, there is an urgent need for a comprehensive study to identify miRNAs or miRNA-regulated mRNAs as prognostic and diagnostic markers. Zhu et al. reported an integrated analysis of the potential roles of the miRNA–mRNA network in TNBC [48]. There is very limited information about the role of miRNA–mRNA interactions in TNBC [48,49], and how miRNAs regulate mRNA expression in TNBC remain elusive. Moreover, the miRNA signatures of Black and White TNBC patients are not well characterized. 

Obesity profoundly alters mRNA expression in TNBC, driving tumorigenesis via multiple molecular pathways. Inflammatory mediators, particularly cytokine signaling components and TNF-pathway genes, are consistently upregulated in obese patients [50]. Likewise, transcripts involved in lipid metabolism and oxidative stress exhibit marked overexpression in TNBC tumors from obese individuals, reflecting a metabolic reprogramming that may fuel tumor progression [51]. In addition, a 24-gene signature has been shown to predict chemotherapy response in TNBC, with potential relevance for obese patients [52]. Despite these advances, and given the known interplay between inflammation, adipocyte biology, and cancer, no study to date has defined an mRNA signature co-expressed with miRNAs in obese TNBC patients [50,51,52].

In this pilot study, we aimed to determine the differential miRNA expression of Black and White TNBC patients with normal weight and obesity compared to their tumor-free adjacent control tissues. Additionally, using bioinformatics analysis, we sought to identify mRNA targets of significant differentially expressed miRNA in obese Black and White TNBC patients.

## 2. Results

### 2.1. Demographics of Patients Included

Samples were randomly selected to establish a balance between race and obesity status. The average age among all cases was 61.4 years, with no significant difference in age distribution between AA and EA cases (mean age 65 vs. 57.7 years; *p* = 0.1474). The stage of disease distribution at diagnosis was classified as local (20.8%), regional (75%), or distant (4.2%), with no significant difference between AA and EA cases (*p* = 1.0000; Table 1).

### 2.2. miRNA Expression Profiles in AA and EA TNBC Patients with or Without Obesity

**Figure 1:**
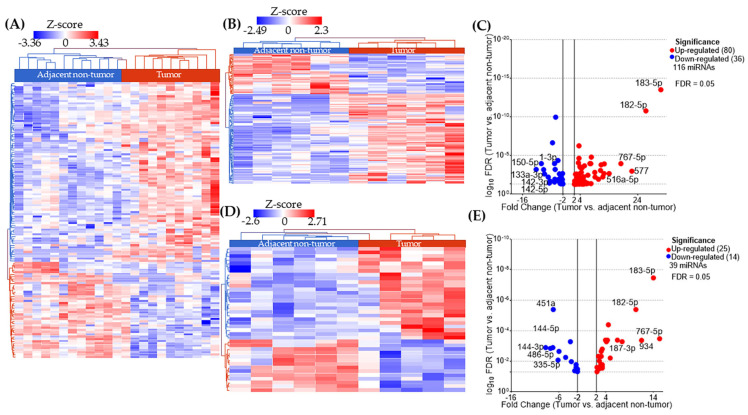
Analysis of differentially expressed (DE) miRNA in tissue samples from European American (EA) patients with triple negative breast cancer (TNBC). (**A**) In total, 124 DE miRNAs separates completely tumor (red bar) from adjacent non-tumor (blue bar) samples in EA women, without regards of the weight status; (**B**) 116 DE miRNAs separates tumor from adjacent non-tumor samples in EA patients with obesity; (**C**) Volcano-plot with labels for the 10 most DE miRNAs; (**D**) 39 DE miRNAs separates tumor from normal samples in EA patients with normal weight; and (**E**) volcano plot with labels for the 10 most DE miRNAs.

**Figure 2:**
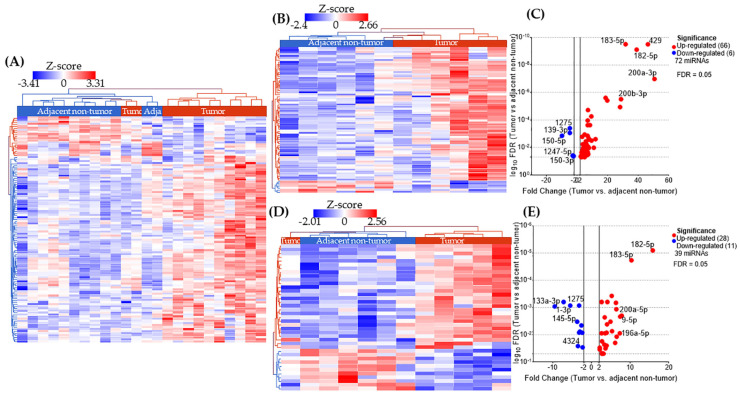
Analysis of differentially expressed (DE) miRNA in tissue samples from African American (AA) patients with triple negative breast cancer (TNBC). (**A**) In total, 109 DE miRNAs separate tumors from adjacent non-tumor samples in AA women, without regard the weight status; (**B**,**C**) 72 DE miRNAs separate tumor from adjacent non-tumor samples in AA patients with obesity and volcano-plot with labels for the 10 most DE miRNAs. (**D**,**E**) In total, 39 DE miRNAs separate tumor from adjacent non-tumor samples in AA patients with normal weight; and volcano-plot with labels for the 10 most DE miRNAs.

We performed miRNA sequencing in 48 FFPE breast tissue samples from women diagnosed with TNBC (24 tumor and 24 adjacent non-tumor tissues). We first performed differential expression analysis (DEA) between tumor and adjacent non-tumor tissues in each racial group (AA and EA), independent of the weight status. The DEA shows 124 and 109 differentially expressed (DE) miRNAs in EA and AA, respectively (Figure 1A, Figure 2A and Figure S1). When we did the analysis based on obesity status in EA patients, we identified 116 DE miRNA between tumor and adjacent non-tumor tissues, with a perfect separation in two clusters in the heatmap (Figure 1B). The top ten DE miRNAs include miR-150-5p, miR-1-3p, miR-133a-3p, miR-142-3p, miR-142-5p, miR-516a-5p, miR-767-5p, miR-577, miR-182-5p, and miR-183-5p (Figure 1C). In the group of lean EA patients, we identified 39 DE miRNAs between tumor and adjacent non-tumor samples, with a perfect separation in two clusters in the heatmap (Figure 1D). The top ten DE miRNAs include miR-144-3p, miR-486-5p, miR-144-5p, miR-451a, miR-335-5p, miR-187-3p, miR-182-5p, miR-934, miR-183-5p, and miR-767-5p (Figure 1E).

The analysis in AA patients, based on obesity status, identified 72 DE miRNAs between tumor and adjacent non-tumor samples, with a perfect separation in two clusters in the heatmap (Figure 2B). The top ten DE miRNAs include miR-150-5p, miR-139-3p, miR-1275, miR-150-3p, miR-1247-5p, miR-200b-3p, miR-183-5p, miR-182-5p, miR-429, and miR-200a-3p (Figure 2C). In the group of lean AA patients, we identified 39 DE miRNAs between tumor and adjacent non-tumor samples, with an almost perfect separation in two clusters in the heatmap (Figure 2D). The top ten DE miRNAs include miR-133a-3p, miR-1-3p, miR-1275, miR-145-5p, miR-4324, miR-196a-5p, miR-200a-5p, miR-9-5p, miR-183-5p, and miR-182-5p (Figure 2E). No adjustments for age, menopausal status, or other clinical covariates were applied due to the limited sample size and the paired design, which inherently controls inter-individual variability.

### 2.3. miRNAs Uniquely Associated with Tumor Tissues in Obese Patients by Racial Group

**Figure 3:**
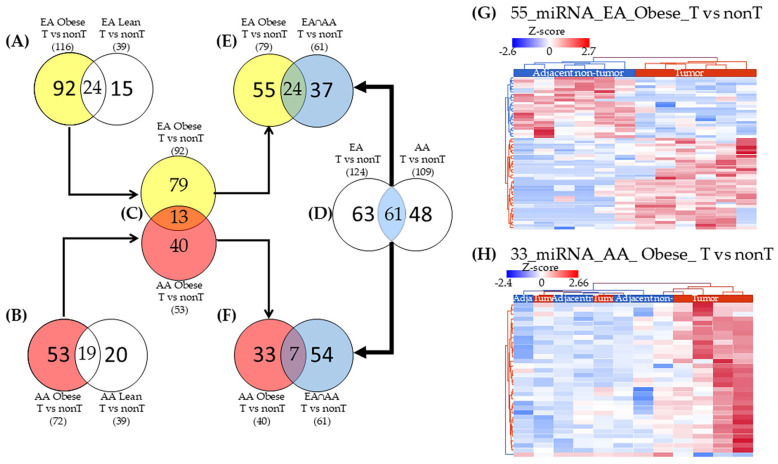
Differentially expressed (DE) miRNAs related to TNBC and obesity in European American (EA) and African American (AA) women. (**A**) In total, 92 miRNAs potentially associated with obesity in tumors versus adjacent non-tumor (T vs. nonT) of EA women (yellow circle); (**B**) 53 miRNAs potentially associated with obesity in T vs. nonT of AA women (red circle); (**C**) 79 miRNAs uniquely related to obese EA (yellow section) and 40 miRNAs with AA (red section); (**D**) 61 common DE miRNAs in Tumors of EA and AA, independent to weight status (blue intersection). (**E**) Unique 55 DE miRNAs in EA women with obesity (yellow section); and (**F**) Unique 33 DE miRNAs in AA women with obesity (red section); (**G**) the 55 DE miRNAs in EA women with obesity distinguish tumors from normal samples; and (**H**) The 33 DE miRNAs in AA women with obesity distinguish partially tumors from normal samples.

To identify the DE miRNAs specifically associated with tumor tissues in obese patients across racial groups, we conducted a comparative analysis between obese and lean patients within each group. Among EA women, comparison of DE miRNAs in obese patients (*n* = 116) versus lean patients (*n* = 39) revealed 92 miRNAs uniquely associated with tumors in obese patients and 15 miRNAs exclusively associated with tumors in lean patients (Figure 3A). Similarly, among AA women, a comparison of DE miRNAs in obese patients (*n* = 72) versus lean patients (*n* = 39) revealed 53 miRNAs associated only with tumors in obese women and 20 miRNAs associated with tumors in lean women (Figure 3B). Direct comparison of tumor expression profiles between obese EA and obese AA patients identified 79 miRNAs unique to EA tumors and 40 miRNAs unique to AA tumors (Figure 3C). To enhance the stringency of the miRNAs selection, we excluded the 61 differentially expressed (DE) miRNAs that were commonly associated with tumor tissues (compared to adjacent non-tumor tissues) in both EA and AA patients, regardless of obesity status (Figure 3D). These miRNAs were removed from the list of 79 miRNAs uniquely associated with tumors in EA patients with obesity (Figure 3E) and from the miRNAs uniquely associated with tumors in AA patients with obesity (Figure 3F). After this refinement, 55 miRNAs remained exclusively associated with tumors from obese EA patients (Figure 3E), and 33 miRNAs were specific to tumors from obese AA patients (Figure 3F). Notably, expression patterns of the 55 EA-specific miRNAs enabled the perfect separation of tumor and adjacent non-tumor tissues in obese EA patients (Figure 3G). In contrast, the 33 AA-specific miRNAs partially distinguished tumor from adjacent non-tumor tissues in obese AA patients (Figure 3H). The differential expression values and identities of these 55 and 33 miRNAs, uniquely associated with TNBC in obese EA and AA women, respectively, are provided in Table S1A,B.

### 2.4. Identification of Differentially Expressed Genes (DEGs) and Correlation with miRNA Expression

**Figure 4:**
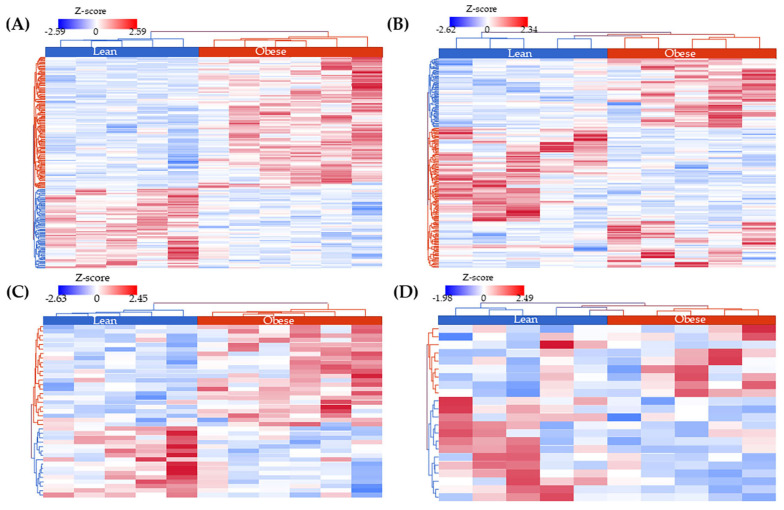
Heatmaps of Differentially Expressed Genes (DEGs) in obese vs. lean patients with triple negative breast cancer (TNBC) by ancestry group. (**A**) All 1,331 DEGs between obese and lean European American (EA) patients. (**B**) All 499 DEGs between obese and lean African American (AA) patients. (**C**) In total, 39 DEGs significantly correlated with miRNA expression in EA patients. (**D**) In total, 22 DEGs significantly correlated with miRNA expression in AA patients. Normalized gene expression values are represented as Z-scores, with red indicating overexpression and blue indicating overexpression. Hierarchical clustering was performed based on Euclidean distance and complete linkage.

With the goal of identifying the transcriptome landscape of tumor tissues of women with obesity (using lean women as reference), we found 499 DEGs in obese AA patients (*p* value ≤ 0.05; 12 with FDR ≤ 0.05) and 1,331 DEGs in obese EA patients (*p* value ≤ 0.05; 21 with FDR ≤ 0.05) (the whole list of genes is found in Table S2). Figure 4A,B show that these DEGs can separate obese from lean tissues from EA and AA patients, respectively.

We performed a post hoc statistical power analysis using the Bioconductor RNASeqPower version 1.48.0 [53]. The analyses included paired and unpaired comparisons of miRNA-seq and mRNA-seq data across EA and AA women (Table S6). For miRNA-seq, the minimal detectable effect sizes ranged from 0.49 to 1.78, with post hoc power estimates between 67% and 97%. Although power was lower in the AA TvsN (regardless of BMI) scenario due to extremely low coverage (average depth ≈ 12 counts), most contrasts achieved power above 80%. This indicates that our sample sizes were sufficient to detect biologically meaningful fold changes in most scenarios, particularly when coverage and variability were favorable (e.g., EA Obese TvsN and EA Lean TvsN). For mRNA-seq, the situation was even more favorable. Minimal detectable effect sizes ranged from 1.78 to 4.74, and post hoc power consistently approached ~100% across contrasts. The substantially higher coverage of mRNA-seq, together with moderate biological variability, ensured that our sample sizes provided an acceptable sensitivity to detect differential expression. We then performed a correlation analysis between normalized counts of DE mRNAs and the unique list of obesity-associated miRNAs in TNBC patients. In EA patients, this included 1331 mRNAs and 55 miRNAs, while in AA, it involved 499 mRNAs and 33 miRNAs. We identified 381 mRNA–miRNA correlations in EA patients and 587 in AA patients, with 41 and 28 showing significant Spearman correlations (*p* ≤ 0.05), respectively (Table S3). Notably, the DEGs correlated with miRNAs demonstrated a strong ability to separate between the obese and lean groups, comparable to the separation observed using all DE mRNAs in each ancestry group (Figure 4C,D).

**Figure 5:**
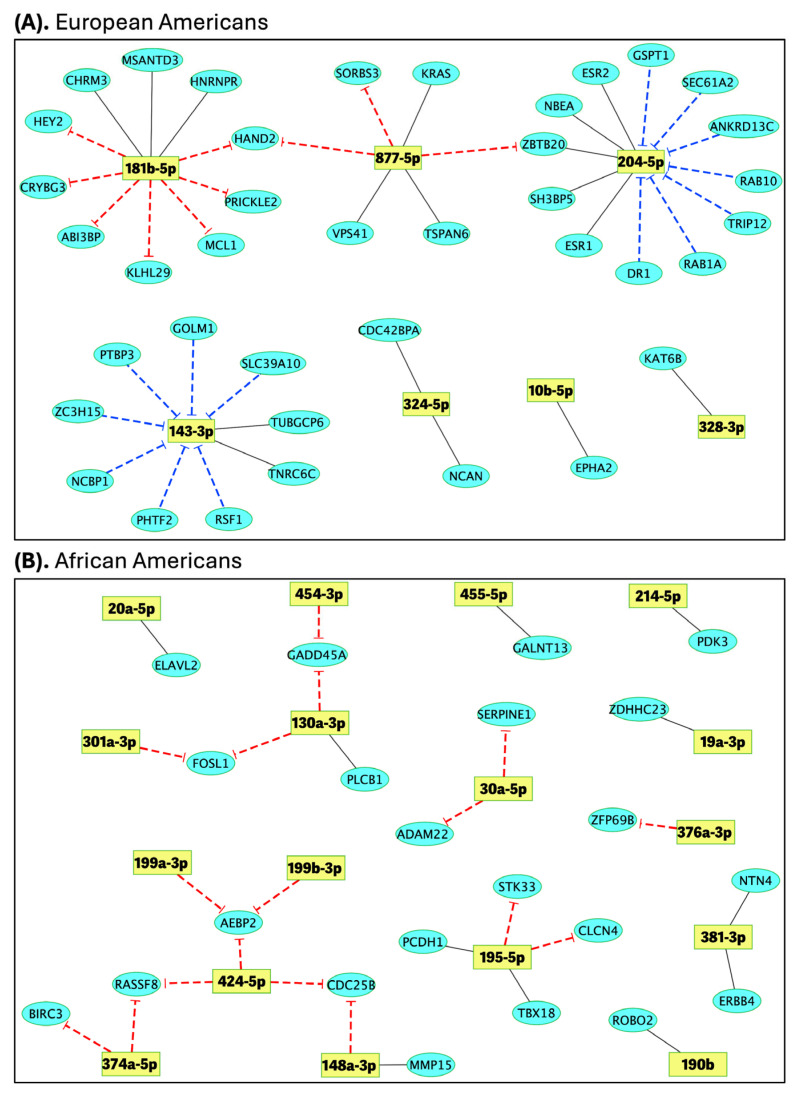
miRNA–mRNA interaction network in obese relative to lean patients in European American (EA) and African American (AA) women. (**A**) In total, 41 significantly correlated interactions, comprising 7 unique miRNAs (yellow nodes) and 39 unique genes (blue edges) in EA. (**B**) In total, 28 significantly correlated interactions, comprising 17 unique miRNAs (yellow nodes) and 22 unique genes (blue edges) in AA. Red dashed inhibition arrows from miRNAs to genes indicate a possible direct interaction mechanism, based on the observed positive fold change in the miRNAs and negative fold change in the genes. Conversely, blue dashed arrows from genes to miRNAs suggest a possible indirect interaction mechanism, based on the observed negative fold change in the miRNAs and positive fold change in the genes.

The integration of differential expression and pairwise correlation analyses, supported by TargetScan-predicted miRNA–mRNA interactions, revealed core regulatory networks in obese TNBC patients (Figure 5). In the EA group, 41 significant miRNA–mRNA interactions were identified, forming a core network of seven miRNAs—miR-143-3p, miR-877-5p, miR-328-3p, miR-204-5p, miR-324-5p, miR-181b-5p, and miR-10b-5p—linked to 39 unique target genes with significant correlations (Figure 5A). In the AA group, the same analytical approach identified 28 significant miRNA–mRNA interactions in obese versus lean patients, involving 17 miRNAs—miR-195-5p, miR-301a-3p, miR-454-3p, miR-130a-3p, miR-148a-3p, miR-424-5p, miR-374a-5p, miR-199a-3p, miR-199b-3p, miR-30a-5p, miR-381-3p, miR-214-5p, miR-376a-3p, miR-190b, miR-19a-3p, miR-455-5p, and miR-20a-5p—connected to 22 unique target genes (Figure 5B).

We focused on the subset of miRNA–mRNA interactions exhibiting inverse correlations (i.e., negative Spearman’s rank correlation coefficients), as these are most indicative of potential regulatory effects. Among the 41 interactions identified in the EA group, 24 showed inverse correlations in obese versus lean TNBC patients. Notably, miR-181b-5p and miR-877-5p were overexpressed in obese patients and demonstrated strong negative correlations with multiple underexpressed target genes. Specifically, miR-181b-5p was inversely correlated with seven genes (*HEY2*, *CRYBG3*, *ABI3BP*, *KLHL29*, *MCL1*, *PRICKLE2*, and *HAND2*), while miR-877-5p was linked to three targets (*HAND2*, *SORBS3*, and *ZBTB20*) (Figure 5A, dotted arrows from miRNA to mRNA). This coordinated expression pattern supports a direct post-transcriptional repression mechanism. In contrast, miR-204-5p and miR-143-3p were underexpressed in obese TNBC patients (negative fold change), yet both displayed significant inverse correlations with upregulated target genes: seven for miR-204-5p (*GSPT1*, *SEC61A2*, *ANKRD13C*, *RAB10*, *TRIP12*, *RAB1A*, and *DR1*) and seven for miR-143-3p (*PTBP3*, *ZC3H15*, *NCBP1*, *PHTF2*, *RSF1*, *SLC39A10*, and *GOLM1*) (Figure 5A, dotted arrows from mRNA to miRNA). This inverse pattern may reflect an indirect regulatory mechanism, such as feedback repression or loss of miRNA-mediated control, potentially indicative of oncogenic target activation. 

On the other hand, among the 28 interactions identified in the AA group, 17 exhibited inverse correlations in obese versus lean TNBC patients. Notably, ten miRNAs were overexpressed in obese patients and demonstrated significant negative correlations with multiple underexpressed target genes, consistent with a direct post-transcriptional repression mechanism. Specifically, miR-195-5p was inversely correlated with *STK33* and *CLCN4*; miR-454-3p and miR-130a-3p with GADD45A; miR-301a-3p with FOSL1; miR-148a-3p and miR-424-5p with *CDC25B*; miR-424-5p and miR-374a-5p with *RASSF8*; miR-374a-5p with *BIRC3*; miR-199a-3p, miR-199b-3p, and miR-424-5p with *AEBP2*; miR-30a-5p with *ADAM22* and *SERPINE1*; and miR-376a-3p with *ZFP69B* (Figure 5B, dotted arrows from miRNA to mRNA). These coordinated inverse expression patterns support a direct inhibitory mechanism of gene regulation in the obese AA TNBC context.

Dashed arrows from miRNAs to genes indicate a possible direct interaction mechanism based on the observed positive fold change in the miRNAs and negative fold change in the genes. Conversely, dashed inhibition arrows from genes to miRNAs suggest a possible indirect interaction mechanism based on the observed negative fold change in the miRNAs and positive fold change in the genes.

### 2.5. Pathways Enrichment Analysis

**Figure 6:**
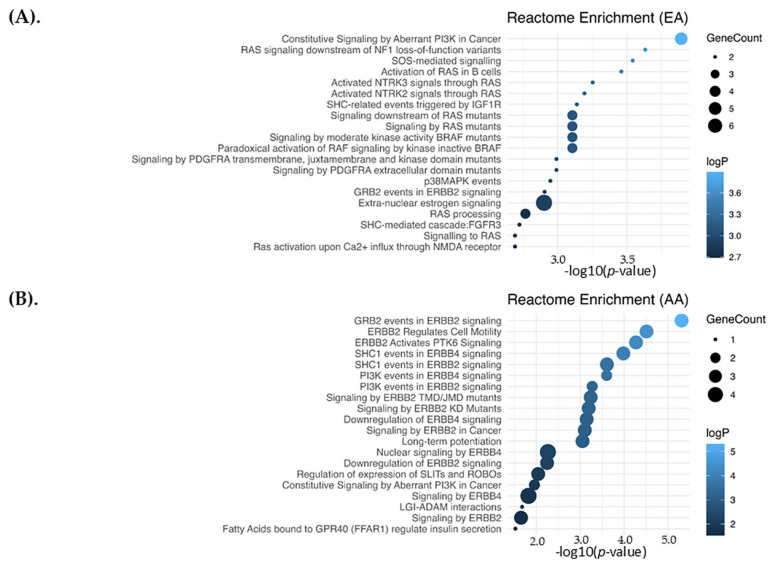
Reactome pathway enrichment analysis for mRNA targets of differentially expressed miRNAs. The top 20 significantly enriched Reactome pathways for correlated mRNA targets of differentially expressed miRNAs in European American (EA) (**A**) and African American (AA) (**B**) groups. Dot size indicates the number of genes involved in each pathway (GeneCount), and color represents the enrichment significance (−log10 of the *p* value).

To investigate the functional relevance of DEGs with significant miRNA correlations, we conducted pathway enrichment analysis using Reactome v92, https://reactome.org, accessed on 1 May 2025. Two gene sets were analyzed: one consisting of 39 DEGs derived from 41 significant miRNA-mRNA correlations in EA patients, and another comprising 22 DEGs derived from 28 significant miRNA-mRNA correlations in AA patients. In the EA group, 90 pathways were significantly enriched (*p* ≤ 0.05), with 11 of the 39 genes (*ESR1*, *ESR2*, *KRAS*, *NCBP1*, *TNRC6C*, *RAB1A*, *RAB10*, *HEY2*, *MCL1*, *GSPT1*, *NCAN*) mapping to at least one pathway, being the mostly represented the Extra-nuclear estrogen signaling and those related with RAS processing (Figure 6A, Table S4). On the other hand, analysis of the AA-specific gene set revealed 27 significantly enriched pathways (*p* ≤ 0.05), with 5 of the 22 genes identified as pathway hits (*ADAM22*, *ERBB4*, *PLCB1*, *ROBO2*, and *SERPINE1*). Notably, in the AA analysis, 12 of the 27 pathways remained significant after FDR correction (adjusted *p* ≤ 0.05), and all these 12 pathways were involved in signaling through *HER2* and *HER4* (Figure 6B, Table S5).

### 2.6. TCGA External Validation

**Figure 7:**
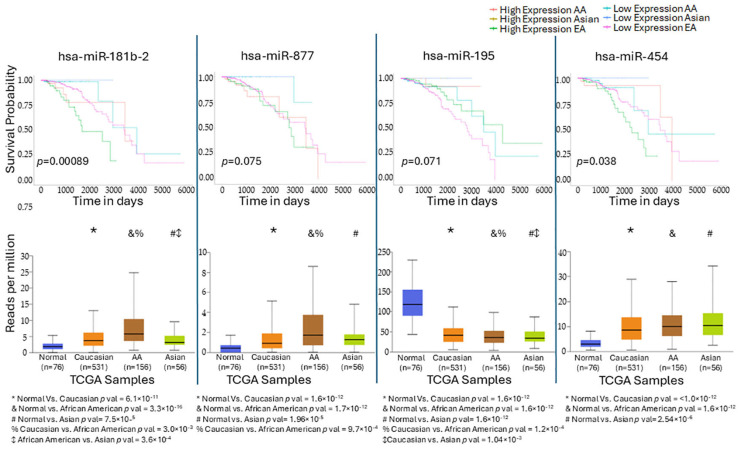
Validation of expression and survival analysis of miRNAs associated with obesity using the TCGA-BRCA database. miR-181b-2 [in European American (EA)] and miR-454 [in African American (AA)] were significantly associated with differential survival in different ethnic groups. Both miRNAs show a significant increase in their expression in tumor tissues, as compared to normal (control) tissues. The expression of the other two miRNAs showed increased (miR-877 in EA) and reduced expression in tumor tissues (miR-195 in AA), but did not show association with survival.

Validation of four candidate miRNAs in the TCGA-BRCA cohort (all breast cancer subtypes) confirmed consistent expression patterns with our dataset. Specifically, miR-181b and miR-877 were overexpressed in tumors from both EA and AA women compared with normal tissues, consistent with our findings in obese versus lean EA women (Figure 7). Interestingly, Kaplan–Meier survival analyses showed that expression of miR-181b is associated with differential survival to breast cancer (*p* = 0.00089), with high expression carrying the worst survival in EA women. Expression of miR-877 was marginally associated with survival (*p* = 0.075) (Figure 7). On the other hand, miR-195 was found to be underexpressed and miR-454 to be overexpressed in AA women relative to normal tissues (Figure 7), in line with our results. miR-454 also showed a significant effect on survival (*p* = 0.038), with low expression conferring the best prognosis in EA women, whereas high expression was linked to poorer outcomes in AA women. Survival differences for miR-195 did not reach statistical significance (*p* = 0.071).

## 3. Discussion

We applied a robust and stringent strategy to identify miRNAs exclusively related to obesity in tumor tissues of AA and EA diagnosed with TNBC. In EA, most of the miRNA–mRNA significative correlated interactions were related to miR-181b-5p, miR-877-5p, miR-204-5p, and miR-143-3p. miR-204-5p functions as a tumor suppressor in breast cancer by inhibiting critical processes such as angiogenesis, invasion, migration, and metastasis. This miRNA was underexpressed almost three times in obese EA tumor samples compared to adjacent non-tumor tissues. This suggests that its targets, such as *SH3BP5*, *NBEA*, *ZBTB20*, *ESR2*, and *ESR1*, may escape miR-204-mediated repression, potentially leading to their overexpression, which could promote tumor growth and metastasis. Similarly, miR-181b-5p, which showed a three-fold increase in obese EA tumor samples, has been reported to be upregulated [54] and to confer doxorubicin resistance in BC [55]. The latter suggests that the mechanism of RNA regulation mediated mainly by these two miRNAs could explain and distinguish TNBC samples between White and Black patients with obesity. miR-877-5p functions as an oncogenic miRNA in TNBC and metabolic disorders by targeting genes such as *IGF2* and *TIMP3*, promoting proliferation and invasion [56]. miR-143-3p acts as a tumor suppressor by regulating *LIMK1* and *MYBL2*, inhibiting cell cycle progression and metastasis in TNBC [57].

In AA, the miR-195-5p, miR-130a-3p, miR-424-5p, miR-148a-3p, miR-199a-3p, miR-199b-3p, miR-301a-3p, miR-30a-5p, miR-374a-5p, miR-376a-3p, and miR-454-3p, in addition to showing significant miRNA–mRNA correlated interactions, also presented inverse fold-change values, suggesting a likely direct or indirect regulatory process. miR-195-5p was significantly overexpressed, while *STK33* was markedly underexpressed, with a strong inverse Spearman correlation. Previous studies have shown that miR-195-5p inhibits proliferation, colony formation, and migration in MDA-MB-231 cells, and causes G1 arrest by directly targeting *CCNE1* [58]. On the other hand, *STK33* has been identified as a survival factor in KRAS-mutant cancers, stabilized by the HSP90/CDC37 complex. Its degradation via HSP90 inhibition induces apoptosis in KRAS-driven cells [59]. The latter suggests a functional axis in which miR-195-5p represses *STK33*, potentially weakening pro-survival signaling in metabolically altered TNBC tumors. This axis could represent a novel regulatory mechanism linking obesity and aggressive tumor behavior. miR-130a-3p was inversely correlated with *GADD45A*, *FOSL1*, and *PLCB1*. *GADD45A* is a well-known tumor suppressor involved in DNA damage response, and its downregulation could impair stress responses [60]. Meanwhile, *PLCB1* promotes radio resistance and immune evasion in TNBC through PI3K/AKT signaling activation, weakening CD8^+^ T cell antitumor responses [61]. miR-424-5p targeted *CDC25B*, *RASSF8*, and, notably, *AEBP2*, a transcription factor and PRC2 cofactor involved in chromatin remodeling and adipogenesis [62]. The repression of *AEBP2* suggests a link between epigenetic regulation and adipocyte-related mechanisms within TNBC. Although evidence of miRNA-mediated regulation of *AEBP2* is limited, recent studies have identified *AEBP2* as a functional target of miR-451a in breast cancer, forming part of a miRNA network that influences proliferation, apoptosis, migration, and invasion [63]. In TNBC, miR-199a 3p acts as a tumor suppressor by targeting BRCA1, impairing DNA repair, and decreasing proliferation and migration—additionally enhancing sensitivity to cisplatin and PARP inhibitors [64]. In obesity, miR-148a-3p is upregulated during adipocyte differentiation, promoting adipogenesis via repression of Wnt1, and its expression correlates positively with BMI in both human and mouse adipose tissues [65]. miR-199b exhibits tumor-suppressive activity in TNBC by inhibiting proliferation and invasion through the downregulation of DDR1 [66]. miR-30a 5p and miR-374a-5p are downregulated in aggressive TNBC, with low levels associated with poorer disease-free and overall survival, underscoring their prognostic potential [47]. Functionally, miR-30a-5p influences epithelial–mesenchymal transition (EMT) and cell cycle pathways. In obesity, miR-30a-5p is notably decreased in the plasma of overweight individuals, linking it to metabolic dysregulation [67]. Conversely, miR-301a-3p is frequently overexpressed in TNBC and associated with poor prognosis; it promotes proliferation, invasion, and metastasis through a feedback loop with cancerous inhibitor of protein phosphatase 2A (Cip2a), an oncogene that is known to inhibit PP2A tumor suppressor activity in human malignancies [47,68,69]. Similarly, miR-454-3p behaves as an oncomir by suppressing tumor suppressors such as PTEN, thereby activating AKT signaling, promoting cell survival, proliferation, migration, and radiation resistance [70]. miR-376a-3p acts as a tumor suppressor in breast cancer by directly targeting neuropilin-1 (NRP-1), leading to the inhibition of Wnt/β-catenin signaling. This suppresses cancer cell proliferation, migration, and invasion while promoting apoptosis [71]. These findings, together with our data, underscore the coordinated repression of genes central to cancer progression, metabolic regulation, and epigenetic control, highlighting ancestry-specific molecular networks in obesity-associated TNBC in AA women.

The observed disparity in the number of significantly correlated miRNA–mRNA interactions between AA and EA patients with TNBC and obesity is a notable finding. It suggests significant implications for treatment and prognosis in AA patients, who have historically received less research attention compared to patients of European ancestry. Limited representation in clinical studies may be one of the reasons AA patients experience worse outcomes despite having a lower incidence of the disease [72]. These heightened miRNA–mRNA interactions in AA patients could lead to the modulation of more unique biological pathways in Black TNBC patients, rendering treatment and prognosis outcomes unfavorable; similarly, an integrative spatial omics study has revealed distinct tumor-promoting microenvironments in AA TNBC patients, which may contribute to poorer treatment responses and less favorable prognoses compared to EA patients [73]. In contrast, the miRNA-regulation in EA TNBC patients with obesity is primarily mediated by two of the miRNAs (miR-204-5p and miR-181b-5p), suggesting that the involved biological and molecular pathways are highly conserved and are most likely involved in known cancer-associated processes.

In TNBC tumors of AA patients with obesity, seventeen miRNAs, seven of which (miR-195-5p, miR-130a-3p, miR-130a-5p, miR-424-5p, miR-148a-3p, miR-374-5p, and miR-30a-5p) potentially downregulated two or more genes (e.g., *CLCN4*, *PLCB1*, *CDC25B*, *AEBP2*, *ERBB4*). However, a conserved scenario was obtained regarding the number of pathways in AA (*n* = 27) when compared to tumors of EA patients with obesity (*n* = 90). Interestingly, eight pathways were shared among TNBC tumors with obesity of both racial groups due to the interactions of different miRNAs of the sets AA and EA with the same related mRNA targets, i.e., *ESR1* and *ES32* DE in EA and *ERBB4* DE in AA, and these belong to the same pathway, ‘Constitutive Signaling by Aberrant PI3K in Cancer’. This is due to a convergence or consensus in key hubs or nodes, of molecular and biological mechanisms that are activated in TNBC, independent of the regulation of miRNAs, suggesting a kind of constitutive mechanisms regulated by different non-miRNAs molecules.

In AA patients, the predominant enriched pathways center on aberrant ERBB2/ERBB4 signaling: GRB2- and SHC1-mediated activation of ERBB2 and ERBB4, PI3K events downstream of both receptors, and constitutive signaling by ERBB2 kinase-domain and transmembrane/junctional mutants. These overlap with cancer-specific ERBB2 and nuclear ERBB4 programs, as well as the downregulation of ERBB2/ERBB4 signaling. Additional modules include regulation of SLIT-ROBO expression and LGI–ADAM interactions, all pointing to dysregulated ERBB family-driven oncogenic signaling. The AA group showed enrichment in ERBB2/ERBB4 signaling pathways (e.g., GRB2, PTK6, PI3K events), despite the TNBC context. These were linked to the receptor-mediated proliferation and regulation of cell motility. Additionally, AA-specific pathways included cytoskeletal remodeling and invasion-related signals (e.g., *ROBO2*, *SERPINE1*, *PLCB1*), pointing to an aggressive, migratory phenotype. Interestingly, PLCB1 induces radioresistance and abates the functional properties of CD8+ T cells in TNBC through the activation of PI3K/AKT pathway [61]. *PLCB1* is overexpressed in adipose tissues of prostate cancer patients [74]. A link between obesity and radioresistance in TNBC and the identification of *SERPINE1* as a determining factor for obesity-associated tumor radioresistance have been reported [75].

In obese EA patients, enriched pathways were dominated by extra-nuclear estrogen signaling (e.g., *ESR1*, *ESR2*, *HEY2*, *MCL1*), suggesting a strong hormonal component in TNBC tumor biology. The most significant process/pathways exclusively found in TNBC tumors of EA patients with obesity are involved mainly with CREB1, and RAS proteins There is documented evidence that CREB1 plays a role in promoting BC [76,77,78]. It has been reported that RAS/MARK activation is associated with the decreased tumor-infiltrating lymphocytes in TNBC [79], suggesting immune evasion in TNBC. RAS-related pathways, including activation, mediation, and downstream RAF/PI3K signaling, indicated proliferative and mitogenic activity, possibly influenced by adiposity. These findings clearly illustrate the underlying differences in gene expression of AA vs. EA TNBC patients with obesity. However, more research is needed to understand how the key regulatory pathways are regulated by miRNA in these two patient cohorts.

We acknowledge that the small sample size of this may limit the statistical power to detect significant genetic associations, and the findings might not fully generalize to larger populations. While we applied False Discovery Rate (FDR) correction to account for multiple testing, no variants remained significant after adjustment, which is expected in studies with limited sample sizes. For this reason, we report unadjusted *p* values while acknowledging the potential for false positives. Future studies should consider increasing the cohort size and employing statistical corrections for multiple testing to strengthen the robustness of the findings and enhance their generalizability. Validation in larger cohorts is necessary to confirm these associations. Because of the paired tumor-adjacent normal design, additional adjustment for age, menopausal status, or other clinical covariates was not required, as the pairing inherently controls inter-individual variability and mitigates potential confounding.

This pilot study is the first to elucidate unique miRNA and mRNA signatures, and their correlations, capable of distinguishing TNBC tumors from obese versus non-obese patients across African American and Caucasian ancestral backgrounds. For this reason, despite extensive efforts, we were unable to identify a publicly available dataset that includes both BMI and racial/ancestral information along with matched transcriptomic data for both miRNAs and mRNAs. As a result, we could not validate our obesity- and race-specific miRNA markers in an independent cohort. Although datasets like TCGA and METABRIC are rich in molecular and survival data, they lack key variables essential to our selection strategy and biological interpretation, specifically BMI data in the case of TCGA and miRNA expression profiles in the case of METABRIC. We acknowledge that this as a limitation; however, it also underscores the novelty and relevance of our study, as it addresses a critical gap in existing resources by integrating obesity and race into the molecular characterization of TNBC for the first time. Future studies incorporating multi-omic data with detailed clinical annotations, including BMI, are needed to build upon these findings and assess their prognostic significance.

One limitation of our pilot study is that race was self-reported and that BMI, while widely used, does not account for body fat distribution or composition—factors that may vary across populations and influence disease biology. To minimize variability and enhance the identification of obesity-related molecular changes, we restricted our analysis to patients with BMI ≥ 30, where metabolic alterations are more consistently observed. This approach strengthened the biological significance of our results, despite the known limitations of BMI and self-reported race.

The miRNA selection strategy is straightforward and rigorous, ensuring that, at each filtering step, we removed miRNAs that could confound interpretation by comparing against appropriate control groups. This approach allowed us to identify miRNAs uniquely associated with obesity-driven TNBC within each race. We then evaluated the correlations between the normalized expression levels of these miRNAs and mRNAs, focusing only on pairs with predicted interactions based on TargetScan, adding a layer of functional relevance. Enrichment analysis of the correlated mRNA targets revealed two distinct biological profiles: immune and inflammatory pathways in AA patients, and metabolic and hormone-related pathways in EA patients. This integrative approach enhances molecular specificity and reveals race-dependent mechanisms through which obesity may influence TNBC biology.

## 4. Materials and Methods

### 4.1. Samples Selection and Study Design

This study was conducted on FFPE tissues acquired and de-identified by the Louisiana Tumor Registry (LTR) after approval by the Institutional Review Board (IRB) at the LSUHSC School of Medicine, which deemed the study as “Exempt”. Figure S1 shows the experimental design for miRNA and mRNA analysis. In brief, de-identified 24 tissues from female BC patients self-identified as African American/Black (AA, *n* = 12) or European American/White (EA, *n* = 12), histologically diagnosed with TNBC and without previous treatment, were randomly and retrospectively selected from a previously funded study (P20CA233374). Within each racial group, 6 patients had obesity (BMI ≥ 30, hereafter referred to as obese), and 6 had normal BMI (18.5–25, hereafter referred to as lean). Forty-eight FFPE samples (24 tumor and 24 adjacent non-tumor tissues) were used to compare the miRNA profiles. Characteristics of the TNBC cases included in this study are shown in Table 1.

### 4.2. RNA Extraction and Library Preparation

All RNA work, including extraction, library preparation, sequencing, and analysis was performed at the Translational Genomics Core (TGC), LSUHSC-New Orleans. Total RNA was extracted from FFPE tissue samples using the truXTRAC FFPE total DNA/RNA (column) kit (Covaris, Woburn, Massachusetts, USA). Directions from the manufacturer were followed, with minor modifications. Briefly, tissue sections of 20 μm were first deparaffinized using xylene substitute (Sigma-Aldrich, St.Louis, USA), followed by two ethanol washes, and were air-dried for 20 min at 37 °C. After deparaffinization, tissues were resuspended in lysis buffer containing proteinase K and lysed using the Covaris M220 focused ultrasonicator (Covaris, Woburn, Massachusetts, USA), following the recommended settings by the manufacturer, followed by incubation at 56 °C for 30 min. The RNA-containing supernatant was then de-crosslinked at 80 °C for 20 min and purified using the RNA purification columns, as indicated by the manufacturer’s instructions. A treatment with DNaseI (QIAGEN, Germantown, MD, USA) was included. RNA was eluted in 30 μl of water. RNA quantification was performed using the Qubit RNA HS Assay kit (Invitrogen, Carlsbad, CA, USA).

Small RNA libraries were generated from 100 ng of total RNA using the QIAseq miRNA library kit and QIAseq miRNA 96 Index IL kit (QIAGEN, Germantown, MD, USA) and according to the manufacturer’s instructions. Briefly, specifically modified adapters were first sequentially ligated to the −3′ and −5′ ends of the RNA, followed by reverse transcription with unique molecular index (UMI) assignment. The cDNA was then amplified using 16 cycles of PCR with a universal forward primer and indexed reverse primers.

mRNA libraries were prepared from tumor samples only, using Illumina’s TruSeq RNA exome library preparation kit (Illumina, San Diego, CA, USA) and according to manufacturer’s instructions, with the following modifications [80]: 300 ng of input RNA was used, PCR cycles were reduced to 9, and hybridization time was extended to 16 hours. After library preparation, free adapters were blocked using Illumina’s free adapter blocking reagent. miRNA and mRNA libraries were validated on Agilent’s 2100 bioanalyzer (Agilent Technologies, Santa Clara, CA, USA) using a High-Sensitivity DNA Kit and quantified using the Qubit dsDNA HS Assay kit (Invitrogen, Carlsbad, CA, USA). For sequencing, libraries were pooled in equimolar ratios and ran on the Illumina’s NextSeq500 (Illumina, San Diego, CA, USA) with single-end 75 bp reads (miRNA) or 2 × 75 bp paired-end (mRNA).

### 4.3. Sequencing Data Processing

miRNA FASTQ files generated with the NextSeq500 instrument were uploaded to GeneGlobe Data Analysis Center of Qiagen^®^ (https://geneglobe.qiagen.com/analyze/, accessed on 22 March 2022). In brief, the trimming of the −3′ adapter and low-quality base was performed using cut-adapt (cutadapt.readthedocs.io/en/stable/guide.html, accessed on 22 March 2022), discarding reads with less than 16 bp insert sequences or less than 10 bp sequences (defective reads), aligning the reads to the reference transcriptome with up to two mismatches tolerated (miRBase V.21, http://www.mirbase.org/, accessed on 22 March 2022) using Bowtie (bowtiebio.sourceforge.net/index.shtml, accessed on 22 March 2022). On average, 7.95 million reads per sample were obtained in the sequencing, and 825,000 of these were mapped to miRBase V.21. A total of 2277 sequences of mature miRNAs were mapped. We used Partek Flow^®^ software (version 12.8.1) perform the miRNA differential expression between tumors and adjacent non-tumor FFPE tissues from obese versus lean EA or AA patients. In brief, the raw counts of UMIs were filtered, keeping miRNAs with at least 5 counts in 80% of the samples, obtaining 1678 miRNAs, which were then normalized using the Trimmed Mean of M-Values (TMM) method, and transformed by log2. DESeq2 algorithm, embedded in Partek Flow, was used to find differentially expressed miRNAs at adjusted *p* values (FDR) of 0.05 and fold change ≥ 2. Heatmaps were made in Partek Flow software, and box and volcano plots in GraphPad Prism 6.0 (San Diego, CA, USA). The miRNA data presented in this pilot study (raw sequencing reads (FASTQ) and normalized reads counts) are openly available for download at the NCBI Gene Expression Omnibus (GEO) under the series accession number GSE279780.

The mRNA samples used in the present work are part of a larger analysis from our laboratory (*n* = 253) deposited in GEO under accession number GSE268851. FASTQ files were obtained from the Illumina’s BaseSpace and were uploaded to Partek Flow, contaminants were removed with Bowtie 2.2.5, aligned to STAR 2.6.1d using hg38 as reference and quantified with RefSeq 96 (release 2 November 2020). Raw counts were filtered to remove features with 5 or less counts in at least 80% of the samples. The trimmed mean of M values (TMM) method was used for normalization, followed by transformation by log2. Differential analysis between samples from obese and lean participants was performed with DESeq2 in Partek Flow using *p* ≤ 0.05 as the cut-off for significance. On average, close to 61 million reads were obtained per sample, with an average depth of 43.4% and coverage of 3.4%.

### 4.4. Strategy for miRNA Selection

To identify miRNAs uniquely associated with tumor tissues in obese patients across racial groups, we implemented a multistep differential expression (DE) filtering strategy (Figure 3). First, within each racial group, EA and AA, we conducted DE analyses comparing tumor versus adjacent non-tumor tissues, stratified by obesity status (obese vs. lean). To isolate miRNAs specifically associated with obesity-related tumors, we excluded any DE miRNAs that were also differentially expressed in lean patients within the same ancestral group. This step ensured the retention of only the miRNAs uniquely deregulated in the context of obesity. Additionally, miRNAs that were differentially expressed in adjacent non-tumor tissues were removed to eliminate background signals not related to the tumor phenotype. To further refine the miRNA lists and remove signals related to shared racial ancestry, we excluded DE miRNAs that were common between EA and AA patients, regardless of obesity status, from the list of miRNAs identified in tumors from obese individuals within each ancestry group (Figure 3D). This comprehensive filtering strategy enabled the identification of ancestry-specific miRNA signatures exclusively associated with TNBC tumors in obese women, while minimizing confounding effects from shared tumor biology or expression changes observed in lean individuals. These refined miRNA sets were then used to investigate downstream target gene interactions and biological pathways potentially mediating the obesity–TNBC relationship.

### 4.5. Correlation Analysis

To explore the regulatory relationships between differentially expressed miRNAs and mRNAs in tumor tissues from obese patients, we performed Spearman rank correlation analysis stratified by ancestry group using Partek Genomics Suite v7.0. Putative miRNA–mRNA interactions were informed by TargetScan v8. Correlations with *p* values ≤ 0.05 were considered statistically significant and retained for downstream analysis. Interaction graphs were plotted in Cytoscape v3.10.3 [81].

### 4.6. Pathway Enrichment Analysis and External Validation in TCGA

To investigate the potential biological functions of the DEGs with significant miRNA associations, we conducted Reactome Pathway Enrichment Analysis (Reactome v92; https://reactome.org, accessed on 1 May 2025). Gene sets derived from the correlation analysis were submitted as input, and pathways with *p* values ≤ 0.05 were considered significantly enriched. FDR correction was applied to account for multiple testing. For external validation, we used the UALCAN portal (http://ualcan.path.uab.edu, accessed on 11 August 2025) to analyze TCGA-BRCA data. Boxplots were generated to compare the expression levels of the candidate miRNAs between tumor and normal breast tissues. In addition, Kaplan–Meier survival analyses were performed to assess the prognostic impact of the candidate miRNAs on overall survival in breast cancer patients.

## 5. Conclusions

Our work highlights the relationship between specific sets of miRNAs, mRNAs, and their correlation, with TNBC in AA and EA individuals in the context of obesity. We identified a robust list of differentially expressed miRNA in tumors of AA and EA TNBC patients with or without obesity, supported by correlation analysis with mRNAs differentially expressed in the same tumor samples and by pathway enrichment analysis. Further analyses are warranted to identify and validate the relevant biomarkers of race and obesity.

## Figures and Tables

**Table 1:** Demographic characteristics of participating patients.

	All	AA	EA	*p* Value
All, *n* (%)	24 (100.0)	12 (100.0)	12 (100.0)	
Age at diagnosis in years, mean (std)	61.4 (13.0)	65.0 (14.0)	57.7 (10.8)	0.1474 ^#^
BMI at diagnosis, *n* (%)				1
Lean	12 (50.0)	6 (50.0)	6 (50.0)	
Obese	12 (50.0)	6 (50.0)	6 (50.0)	
Tumor stage (SEER), *n* (%)				1
Localized	5 (20.8)	3 (25.0)	2 (16.7)	
Regional	18 (75.0)	9 (75.0)	9 (75.0)	
Distant	1 (4.2)	0 (0.0)	1 (8.3)	

Abbreviations: BMI = body mass index, SEER = Surveillance, Epidemiology, and End Results program; # Student’s *t*-test.

## Data Availability

The data generated in this study are available at the Gene Expression Omnibus under accession number GSE279780 and GSE268851.

## Supplementary Materials

The following supporting information can be downloaded at: https://www.mdpi.com/article/10.3390/ijms26189101/s1.

## Abbreviations

White/EA	European American
Black/AA	African American
DE	Differentially Expressed
DEA	Differential Expression Analysis
miRNAs	microRNAs
FFPE	Formalin-fixed paraffin-embedded
TNBC	Triple Negative Breast Cancer
BC	Breast Cancer
FDR	False discovery rate
TGC	Translational Genomics Core
