# Supplementary material for: Integrated miRNA-mRNA Analyses of Triple-Negative Breast Cancer in Black and White Patients with or Without Obesity
Source: Int J Mol Sci. 2025 Sep 18;26(18):9101. doi: 10.3390/ijms26189101 (PMC12471265; doi:10.3390/ijms26189101)
Supplement: Supplementary file 1 [file ijms-26-09101-s001.zip › ijms-3876045-suple-figure.pdf]

Supplementary Materials:

Supplementary Table S1. (A). Signature of unique, differentially expressed miRNAs in obese TNBC patients in EA. **(B).** Signature differentially expressed mRNAs in obese TNBC patients in AA.

Supplementary Table S2. Signature of differentially expressed mRNAs in obese TNBC patients in EA **(A)** and AA **(B).**

Supplementary Table S3. Spearman correlation between predicted miRNA–mRNA interactions among the differentially expressed genes. **(A)** EA; **(B)** AA.

Supplementary Table S4. Pathway enrichment analysis of mRNAs differentially expressed in EA with significant correlations to miRNAs.

Supplementary Table S5. Pathway enrichment analysis of mRNAs differentially expressed in AA with significant correlations to miRNAs.

Supplementary Table S6. Post-hoc statistical power calculations across scenarios in miRNA-seq and RNA-seq datasets.

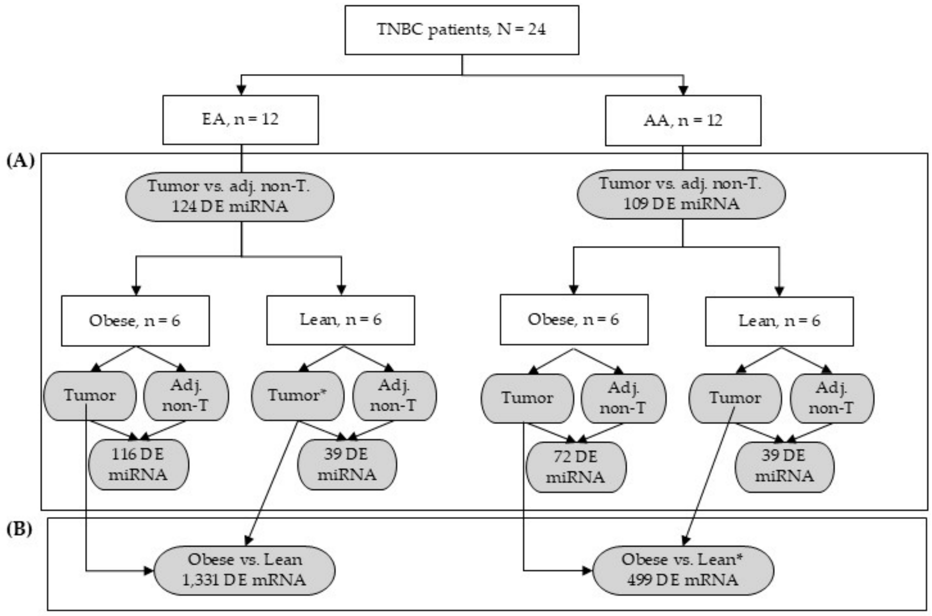

Supplementary Figure S1. Distribution of TNBC patients and summary of differential expression analyses by ancestry and obesity status. A total of 24 TNBC patients were included, equally divided into European American (EA, n = 12) and African American (AA, n = 12) groups. Each ancestry group was further stratified by weight status into obese (n = 6) and lean (n = 6) subgroups. **(A)** miRNA-seq analysis: Differential expression analysis (DEA) was performed between tumor and matched adjacent non tumor tissues for each subgroup. The number of differentially expressed (DE) miRNAs is indicated per comparison. **(B)** mRNA-seq analysis: Conducted on tumor tissue only, comparing obese versus lean patients within each ancestry group. A total of 1,331 DE mRNAs were identified in EA patients and 499 in AA patients. *One tumor sample from EA lean group did not pass library quality control for miRNA-seq, and one tumor sample from the EA obese group did not pass library quality control for mRNA-seq.
